# Old-age-onset subconjunctival juvenile xanthogranuloma without limbal involvement

**DOI:** 10.1186/1471-2415-14-24

**Published:** 2014-03-06

**Authors:** Mo-Sae Kim, So-Ah Kim, Ho-Seok Sa

**Affiliations:** 1 Department of Ophthalmology, Asan Medical Center, University of Ulsan, College of Medicine, 388-1 Pungnap-2-dong, Songpa-gu, 138-736 Seoul, Korea

**Keywords:** Juvenile, Xanthogranuloma, Subconjunctival

## Abstract

**Background:**

Juvenile xanthogranuloma (JXG) is a benign idiopathic cutaneous granulomatous tumor occurring primarily in infants less than 1 year old, and less commonly found in older children and adults. To date, however, there have been no reports of patients aged >50 years with cornealscleral JXG without limbal involvement. We describe here a 58-year-old woman with subconjunctival JXG without limbal involvement.

**Case presentation:**

A 58-year-old female was referred for evaluation of a subconjunctival mass in her left eye, found incidentally 2 weeks earlier. Examination revealed a protruding yellow-orange subconjunctival mass just below the 6-o’clock limbus of her left eye, measuring 6.0 × 4.5 mm, but not extending into the cornea. The overlying conjunctival epithelium was intact, and a feeding vessel was observed between the mass and the episclera. The subconjunctival lesion was excised under local anesthesia, by dissecting the mass from the overlying conjunctiva and underlying sclera. The conjunctiva was reattached to the sclera without creating a bare area. Hematoxylin and eosin-stained sections showed that the mass was a mixed inflammatory lesion containing dense infiltrations of epithelioid histiocytes with foamy cytoplasm, lymphocytes, and plasma cells, as well as multinucleated Touton giant cells with the characteristic circumferential ring of nuclei. Immunohistochemical staining showed that the lesion was positive for the macrophage marker CD68 and negative for the Langerhans cell markers S-100 protein and CD1a, indicating that the lesion was a xanthogranuloma. The patient has been followed up for 12-months without recurrence.

**Conclusions:**

JXG can occur as a solitary subconjunctival mass even in older adults, and immunohistochemistry is useful in differential diagnosis. Simple excision with careful dissection may be effective for subconjunctival JXG.

## Background

Juvenile xanthogranuloma (JXG) is a benign idiopathic cutaneous granulomatous tumor occurring primarily in infants less than 1 year old, and less commonly found in older children and adults [1,2]. Cutaneous lesions appear as orange-red macules or papules, predominantly over the face, neck, and upper trunk and usually resolve spontaneously over 1 to 5 years [1]. Ocular JXG occurs up to 10% of patients with JXG, usually as a solitary mass in the iris. This can cause spontaneous hyphema or secondary glaucoma, threatening the vision of affected individuals [1,3]. Ocular JXG may also involve the eyelid, corneoscleral limbus, conjunctiva, orbit, retina, choroid, disc, and optic nerve [2,4,5].

Although JXG mainly occurs in infants, it is occasionally encountered in adults, with several adults reported with corneoscleral limbal JXG [6-11]. To date, however, there have been no reports of patients older than 50 years of age with corneoscleral JXG without limbal involvement. Here, we describe a 58-year-old woman who presented with subconjunctival JXG without limbal involvement.

## Case presentation

This study has been granted an exemption from requiring ethics approval by the Institutional Review Board of the Ethics Committee of Asan Medical Center, Seoul, Korea, under the tenets of the Helsinki declaration.

**Figure 1:**
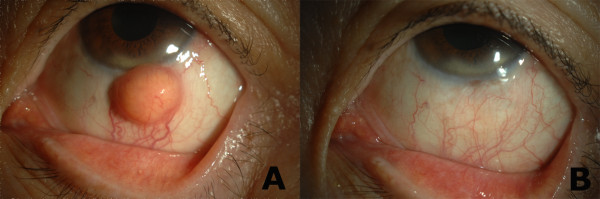
**Anterior segment findings. (A)** A yellow-orange subconjunctival mass with feeding vessels below the 9 o/c limbus. The overlying conjunctiva was intact. **(B)** Twelve months postoperatively, there was no evidence of recurrence.

A 58-year-old female was referred for evaluation of a subconjunctival mass in the left eye found incidentally 2 weeks earlier. The patient’s medical history was unremarkable, with no individual or family history of ocular disease. Ocular examination showed a protruding yellow-orange subconjunctival mass just below the 6-o’clock limbus of her left eye, measuring 6.0 × 4.5 mm without extending into the cornea (Figure 1A). The overlying conjunctival epithelium was intact, and a feeding vessel was observed between the mass and the episclera. Orbital computed tomography with enhancement and ocular examination showed no evidence of skin, periorbital, iris, or posterior segment involvement. Her left eye had an uncorrected visual acuity of 20/25, an intraocular pressure of 14 mmHg, and an autokeratometric cylinder of − 0.5 D with an axis of 155 degrees.

The subconjunctival lesion was excised under local anesthesia by dissecting the mass from the overlying conjunctiva and underlying sclera. The conjunctiva was reattached to the sclera without creating a bare area.

**Figure 2:**
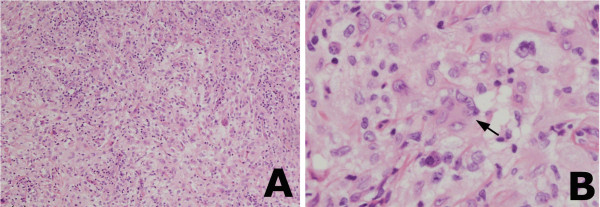
**Histological findings. (A)** Hemotoxylin-eosin staining, showing a mixed inflammatory lesion composed of dense infiltrates of epithelioid histiocytes with foamy cytoplasm, lymphocytes, plasma cells, and multinucleate giant cells (×100). **(B)** Multinucleate Touton giant cells with the characteristic circumferential ring of nuclei (arrow), surrounded by histiocytes (×200).

**Figure 3:**
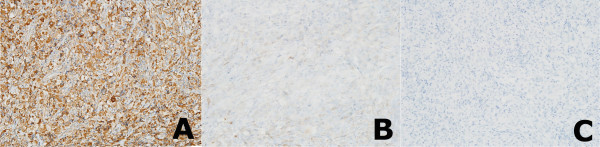
**Immunohistochemical stainings.** Strong positive staining for the macrophage marker CD68 **(A)**, and negative staining for the Langerhans cell markers S-100 protein **(B)** and CD1a **(C)** (×100).

Hematoxylin and eosin-stained sections showed that the mass was a mixed inflammatory lesion, with dense infiltration of epithelioid histiocytes with foamy cytoplasm, lymphocytes, and plasma cells, as well as multinucleated Touton giant cells characterized by a circumferential ring around the nuclei (Figure 2). Immunohistochemical staining showed that the lesion was positive for the macrophage marker CD68 and negative for the Langerhans cell markers S-100 protein and CD1a, indicating that the lesion was a xanthogranuloma (Figure 3).

The patient has been followed up closely for 12-months, with no sign of recurrence (Figure 1B).

## Discussion

The differential diagnosis of a yellowish conjunctival mass, with or without limbal involvement, includes epibulbar dermoid, dermolipoma, and, less frequently, phlyctenular keratoconjunctivitis, neurofibroma, fibrous histiocytoma, pterygium, pyogenic granuloma, and foreign body granuloma, as well as other primary and secondary inflammatory lesions such as JXG and Langerhans cell histiocytosis [2]. Histopathologically, the typical findings of JXG include a mixture of foamy and epitheloid histiocytes with scattered lymphocytes, eosinophils, and plasma cells, and typical Touton giant cells with wreath shaped nuclei [1]. Occasionally, corneoscleral limbal JXG must be differentiated from other, more severe conditions, such as Langerhans cell histiocytosis [12]. Most JXG lesions are positive for macrophage markers, such as CD 68 and HAM 56 [2,11], but are negative for Langerhans cell markers, such as S-100 and CD1a [13]. S-100, however, may be positive; for example, a recent series found that 6 of 100 cutaneous JXGs were positive for monoclonal markers of S-100 protein [14]. The yellowish subconjunctival lesion in our patient was positive for CD68 but negative for S-100 and CD1a, findings compatible with a diagnosis of JXG.

Cutaneous JXG usually improves spontaneously without any treatment. Ocular lesions, however, rarely disappear spontaneously and thus require treatment. Although there is no standard treatment for ocular JXG, several modalities have been tried, including surgical excision, intralesional steroid injection, cryotherapy, and low dose radiotherapy [2,11,15]. Patients with lesions recurrent or resistant to these treatments may be treated with systemic chemotherapy [13,16]. In most previous patients, corneoscleral JXG was not associated with any systemic xanthogranulomatous disease, suggesting that limbal JXG is a localized disorder. Most patients with limbal JXG have been successfully treated by simple excision, with or without keratectomy and lamellar graft. Since the mass in our patient did not involve the cornea, simple excision without keratectomy was sufficient to control for recurrence.

The patient described here can be distinguished from previous patients with ocular JXGs. To our knowledge, our patient, at the age of 58, is the oldest reported to date among patients with ocular JXGs [6-11]. In addition, this is the second report of a rare solitary subconjunctival JXG without limbal involvement. The first patient, a 43-year-old man, was treated with excision and additional cyrotherapy without recurrence for 2 months [11]. By contrast, our patient was successfully treated by simple excision, without recurrence for 1 year.

## Conclusion

JXG can occur as a solitary subconjunctival mass even in older adults, and immunohistochemistry is useful in differential diagnosis. Simple excision with careful dissection may be effective in treating a subconjunctival JXG.

## Consent

Written informed consent was obtained from the patient for publication of this case report and any accompanying images. A copy of the written consent is available for review by the Editor of this journal.

## Competing interests

The authors declare that they have no competing interests.

## Authors’ contributions

Conception and design: HSS, Analysis and interpretation of data: HSS, MSK, SAK, Drafting the article or revising it critically for important intellectual content: MSK, HSS, Final approval of the version to be published: HSS. All authors read and approved the final manuscript.

## Pre-publication history

The pre-publication history for this paper can be accessed here:

http://www.biomedcentral.com/1471-2415/14/24/prepub
